# Small and Oriented Wheat Spike Detection at the Filling and Maturity Stages Based on WheatNet

**DOI:** 10.34133/plantphenomics.0109

**Published:** 2023-10-30

**Authors:** Jianqing Zhao, Yucheng Cai, Suwan Wang, Jiawei Yan, Xiaolei Qiu, Xia Yao, Yongchao Tian, Yan Zhu, Weixing Cao, Xiaohu Zhang

**Affiliations:** ^1^ National Engineering and Technology Center for Information Agriculture, Nanjing Agricultural University, Nanjing 210095, China.; ^2^ Key Laboratory for Crop System Analysis and Decision Making, Ministry of Agriculture and Rural Affairs, Nanjing 210095, China.; ^3^ Jiangsu Key Laboratory for Information Agriculture, Nanjing 210095, China.; ^4^ Jiangsu Collaborative Innovation Center for Modern Crop Production, Nanjing 210095, China.

## Abstract

Accurate wheat spike detection is crucial in wheat field phenotyping for precision farming. Advances in artificial intelligence have enabled deep learning models to improve the accuracy of detecting wheat spikes. However, wheat growth is a dynamic process characterized by important changes in the color feature of wheat spikes and the background. Existing models for wheat spike detection are typically designed for a specific growth stage. Their adaptability to other growth stages or field scenes is limited. Such models cannot detect wheat spikes accurately caused by the difference in color, size, and morphological features between growth stages. This paper proposes WheatNet to detect small and oriented wheat spikes from the filling to the maturity stage. WheatNet constructs a Transform Network to reduce the effect of differences in the color features of spikes at the filling and maturity stages on detection accuracy. Moreover, a Detection Network is designed to improve wheat spike detection capability. A Circle Smooth Label is proposed to classify wheat spike angles in drone imagery. A new micro-scale detection layer is added to the network to extract the features of small spikes. Localization loss is improved by Complete Intersection over Union to reduce the impact of the background. The results show that WheatNet can achieve greater accuracy than classical detection methods. The detection accuracy with average precision of spike detection at the filling stage is 90.1%, while it is 88.6% at the maturity stage. It suggests that WheatNet is a promising tool for detection of wheat spikes.

## Introduction

Wheat is one of the world’s most important food crops [1]. With the development of remote sensing technology, collecting data from various remote sensing platforms has been recognized as an effective technical solution to support crop production decisions. Detecting and characterizing wheat spike individuals from remote sensing images facilitate the recording of the wheat growth, which is important for field management [2]. With the convenience and practicality of unmanned aerial vehicles (UAVs), detecting and counting wheat spikes have attracted much interest from researchers.

Wheat growth is a continuous and dynamic process that occurs over time. Monitoring wheat growth over several stages can provide a more accurate wheat yield prediction [3]. Color characteristics are considered to be one of the most recognizable and discernible characteristics by humans. These characteristics can effectively describe the variations in wheat spike color and background differences [4]. Combined with color, texture, and geometric features, Bayesian, Support Vector Machine, Random Forest, and other matching learning algorithms are used to detect and segment wheat spikes [5,6]. Among these features, color is essential for detecting and counting wheat spikes [7,8]. As a data-driven approach, the features obtained from deep learning are more abstract, robust, and generalizable. In recent years, wheat spike detection methods have made progress due to the rapid development of deep learning. Researchers have analyzed wheat spike images and proposed convolutional neural networks for wheat spike counting, including classification, detection, and segmentation [5,8–10]. In addition to classification and segmentation, detection methods are designed to detect individuals and are convenient for counting spikes [11]. Two-stage and one-stage networks are the 2 main methods of object detection. Two-stage networks divide the detection task into 2 steps. First, proposals containing object information are generated by the region proposal network. Second, the location, category, and confidence of objects can be predicted from the proposals. Based on these processes, Faster Region-based Convolutional Neural Network (Faster R-CNN) [12], Region-based Fully Convolutional Network (R-FCN) [13], Efficientnet [14], and other methods are designed to improve accuracy. Compared with 2-stage networks, a 1-stage network is faster because it makes predictions without generating proposals. One-stage networks mainly include RetinaNet [15], Single Shot Detector (SSD) [16], and the You Only Look Once (YOLO) models [17–20].

Like other object detection methods, the color of wheat spikes in drone imagery is influenced by the growth stage of wheat and dominates the performance of wheat spike detection methods [5,21]. This observation highlights that color-based methods for wheat spike detection are often used for specific growth stages. To overcome this challenge, several strategies have been adopted to detect wheat spikes at different stages accurately. The first approach is to develop different wheat spike detection models for specific stages. While this approach can achieve higher accuracy, it can be less efficient and has limited applicability [22,23]. The second approach is to develop a single wheat spike detection model trained on uniform wheat spike images across all growth stages, using the same model parameters for feature extraction. However, a single model is challenging as it requires a large amount of training data with images, and including all the different characteristics of wheat spikes from different stages may not be feasible. Variations in the image data can lead to discrepancies in the category distribution, ultimately reducing the model’s accuracy [24]. The third approach is to develop a wheat spike detection model with transfer learning for a specific growth stage [25]. The transfer ability of the model is affected by variations in wheat spike characteristics. In addition, with the powerful automatic feature extraction capabilities of convolutional neural networks, recent researchers have adapted methods from other domains and focused on optimizing network structures. These proposed neural networks are generally composed of 2 parts: the first part is the pre-trained module, which is used as a basic feature extractor; the other is an optimized module for the specific task [26–29]. It provides guaranteed methods for accurate classification and detection with less training images [30]. However, these approaches ignore existing agronomic knowledge and do not thoroughly investigate the differences in color characteristics of wheat spikes at different growth stages and their effect on detection accuracy. Furthermore, the small size, dense distribution, and severe occlusion of wheat spikes in drone imagery also make it difficult for the approach to fit and cover all the features of wheat spikes, resulting in low accuracy and poor applicability.

With our previous work [31,32], this paper constructs WheatNet to detect small and oriented wheat spikes in drone imagery from the filling to the maturity stage to solve the abovementioned issues. WheatNet combines the color, orientation, and size characteristics of wheat spikes that can accurately detect wheat spikes at different growth stages and is suitable for field scenes. Data augmentation in WheatNet is adopted to preprocess the images of wheat spikes captured by the UAV. WheatNet consists of 2 sub-networks: a Transform Network and a Detection Network. The former is responsible for image transformation and generating new wheat spike images by fusing color features. At the same time, the latter can generate multi-scale feature maps and predict the location, size, and category of wheat spikes. WheatNet provides accurate detection on small and oriented wheat spikes, making it more suitable for use in complex field environments.

## Materials and Methods

### Experimental design and data collection

**Fig. 1.**
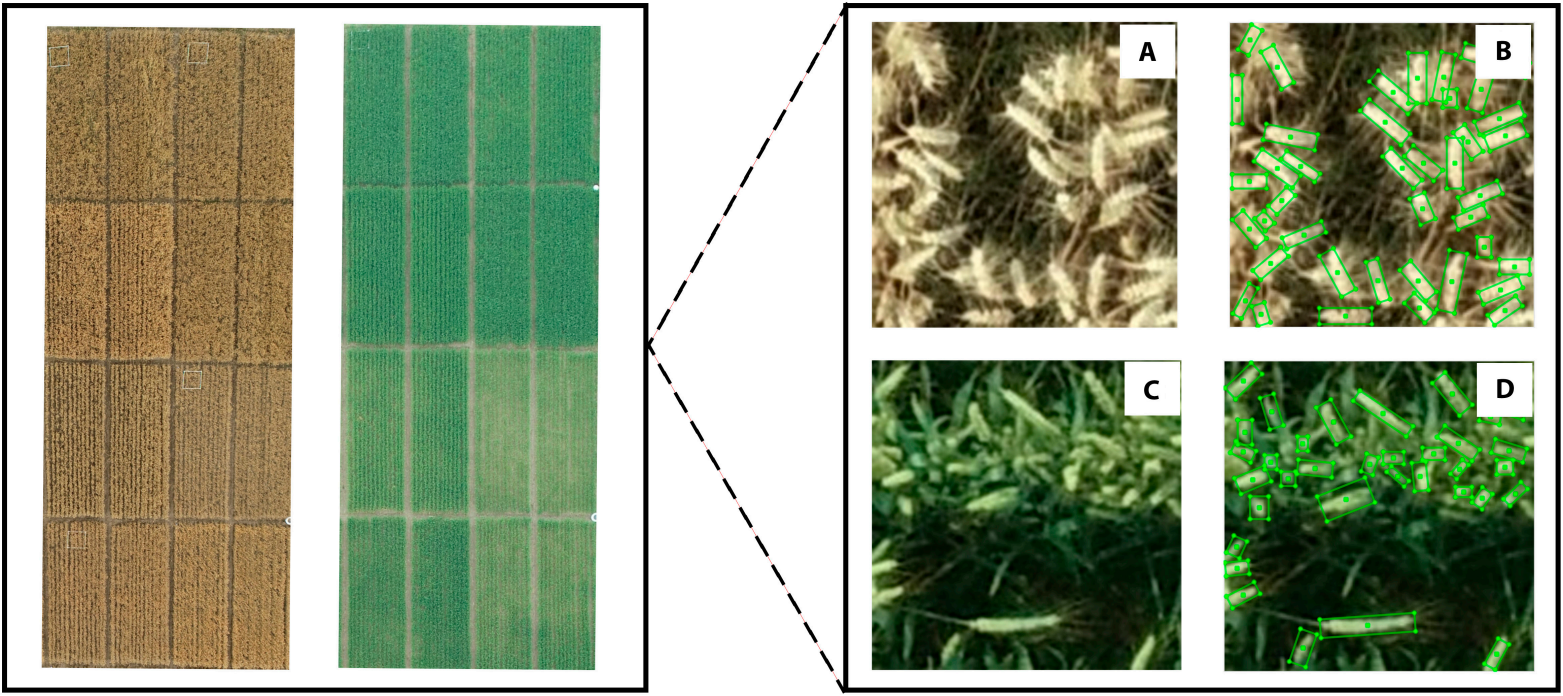
The experimental field and UAV images of wheat spikes with annotation results. (A) UAV images captured at the maturity stage, (B) annotation results of the corresponding image at the maturity stage, (C) UAV images captured at the filling stage, and (D) annotation results of the corresponding image at the filling stage.

The experiment was conducted at Rugao City in Jiangsu Province. Eight local wheat cultivars such as Huaimai 33, Jimai 22, Yangmai 23, Ningmai 13, Yangfumai 4, Yannong 19, Yangmai 16, and Zhenmai 12 were planted in 16 plots of 6 m × 6 m. Visible images were captured with a DJI ZENMUSETM X4S camera at 10 m high. We conducted the flying task at the filling and maturity stages. The UAV images were captured between 10:00 AM and 2:00 PM to minimize variations in lighting and mitigate the impact of shadows. A total of 300 images were acquired, with 150 images obtained at the filling stage and 150 images obtained at the maturity stage. One visible image was cropped into 4 small images with a 150 × 150 pixel resolution to improve data processing efficiency, highlight spike characteristics, and facilitate convolutional network training. The area of each cropped image was 0.25 m^2^. These small images were then labeled with the image annotation tool RoLabelImg [33] (Fig. 1).

Data augmentation, including image flipping, image rotation, and brightness balance, was adopted to expand the capacity of the dataset and to enhance the generalization ability of the model. Brightness balance can eliminate the effect on detection performance caused by illumination variations and different sensors [31]. Each small image was augmented 8 times and 7,000 images were generated after data augmentation. They were randomly selected and split into a training set, a validation set, and a test set in a 7:2:1 ratio.

### WheatNet for wheat spike detection

**Fig. 2.**
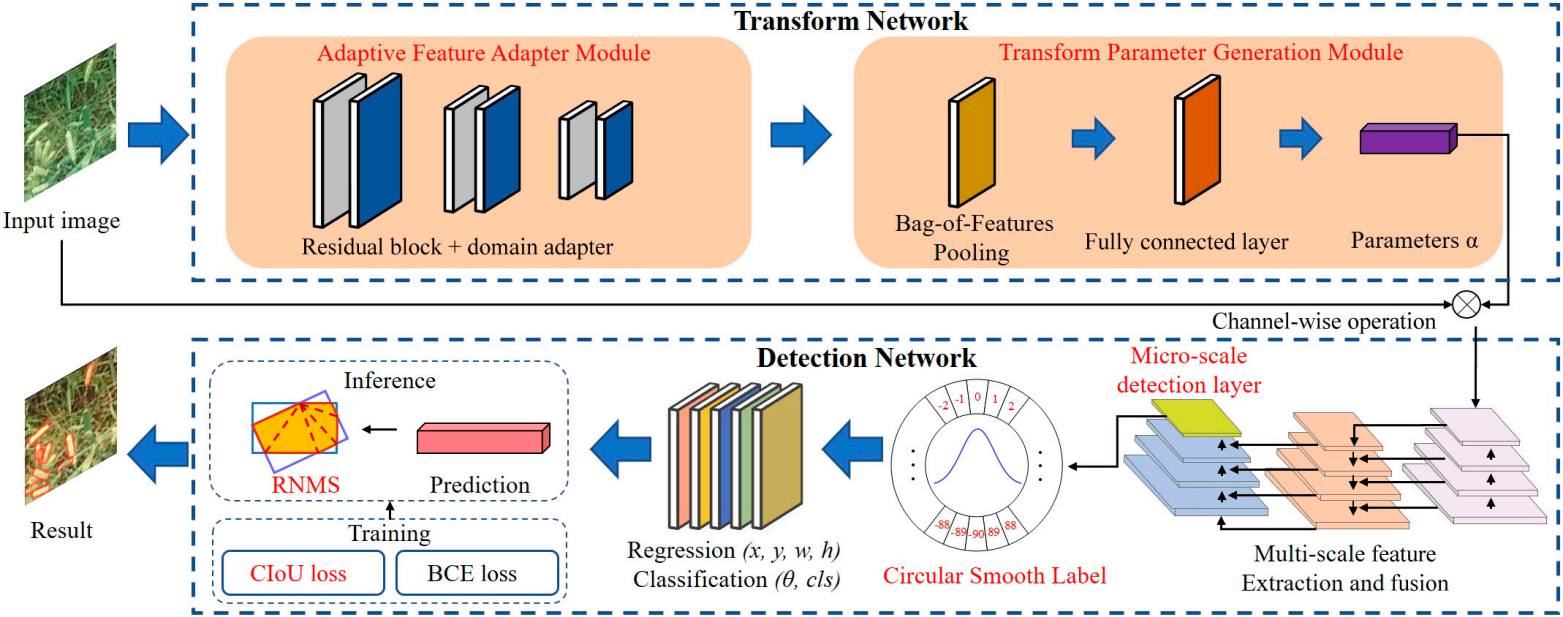
WheatNet architecture. The improved modules with red labels are the micro-scale detection layer, Circular Smooth Label (CSL), and Complete Intersection over Union (CIoU) loss.

WheatNet is a one-stage detection method that can dynamically generate transformation parameters to reduce the variation in wheat spike characteristics between growth stages. It can accurately detect wheat spikes at the filling and maturity stages. The architecture of WheatNet is shown in Fig. 2. WheatNet consists of 2 sub-networks: a Transform Network and a Detection Network. First, the Adaptive Feature Adapter Module (AFAM) in the Transform Network is proposed to generate weights sensitive to wheat spikes’ color at different growth stages. Second, the Transform Parameter Generation Module (TPGM) uses a Bag-of-Features Pooling (BOFP) layer to learn the color features of wheat spikes and output transformation parameters *α* to generate the new image with a fully connected layer. Third, the Detection Network extracts multi-scale features from the new image. Then, a micro-scale detection layer is added to the Detection Network, and 4 detection layers generate predictions, including position, category, and confidence of wheat spikes from multi-scale feature maps. At this stage, a Circle Smooth Label (CSL) is used to classify angles for wheat spikes. Complete Intersection over Union (CIoU) loss and Binary Cross Entropy (BCE) loss are used to calculate the network loss for training. Finally, detection results are produced from prediction during the inference after training.

### Transform Network

The color information of wheat varies significantly during growth, and illumination and background can cause differences in color features of wheat spikes between the filling and maturity stages. It makes the methods rely on color information on wheat spike detection typically only applicable to specific growth stages [6,25]. In this study, a Transform Network is introduced to solve this issue. The Transform Network dynamically generates linear color transformation parameters to reduce the variation of wheat spike features at different growth stages, providing the Detection Network with new wheat spike images.

The Transform Network includes an AFAM and a TPGM. AFAM utilizes attention mechanisms to learn weights sensitive to wheat spike colors during the filling and maturity stages. TPGM combines these weights with a BOFP layer and a fully connected layer to generate transformation parameters dynamically. These parameters are used to transform values of the image’s R, G, and B bands to reduce the effect of differences in wheat spike color features between growth stages.

**Fig. 3.**
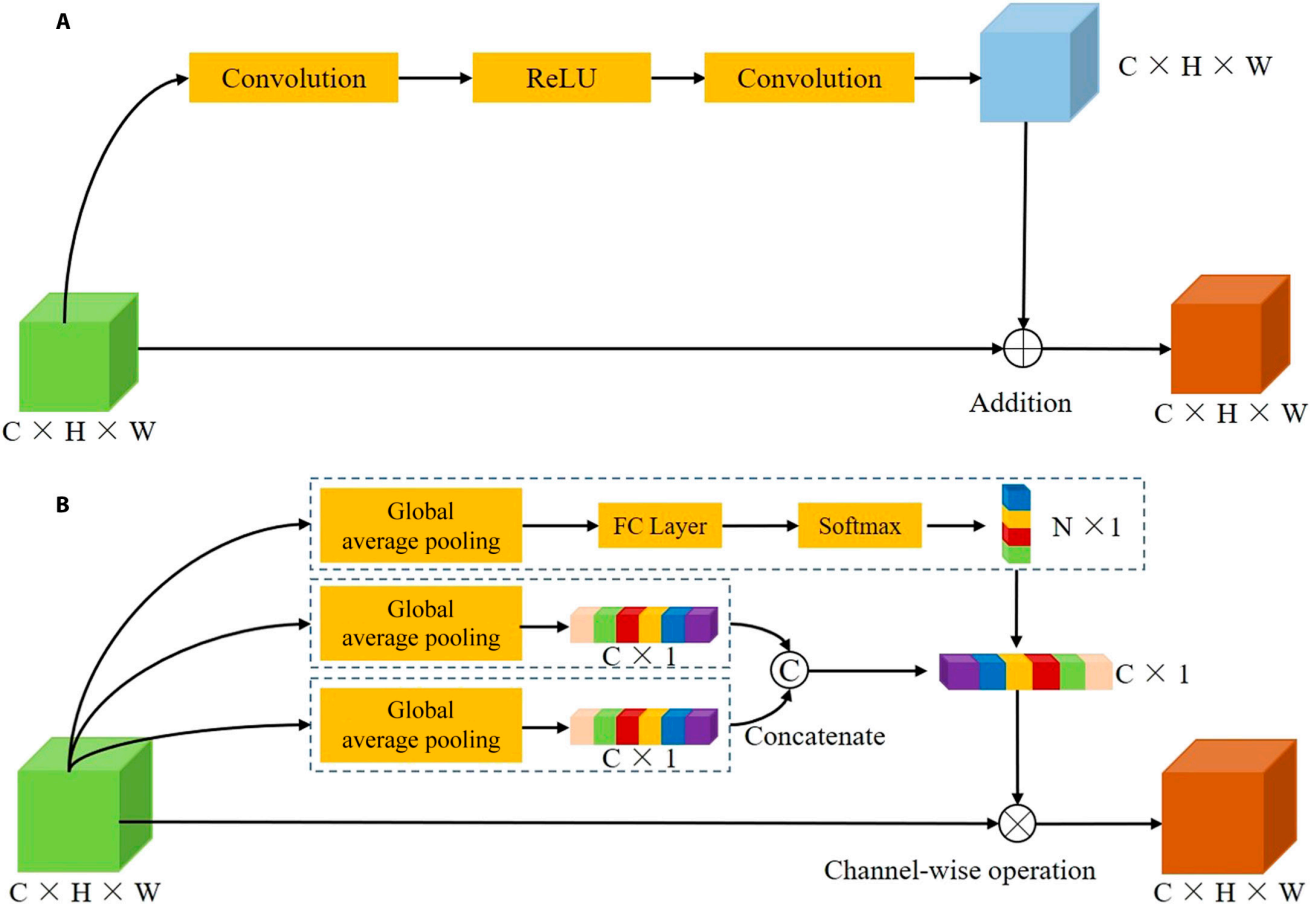
The structure of the Adaptive Feature Adapter Module: the residual block layer (A) and the domain adapter layer (B).

Inspired by the ResNet network and attention mechanism [34,35], AFAM contains residual block layers and domain adapter layers (Fig. 3A and B). Multi-scale features from wheat spike UAV images are extracted, and the skip connection of residual block layers helps to avoid gradient disappearance and explosion in convolutional networks. Each residual block layer consists of 2 convolutional layers and a Rectified Linear Unit (ReLU) activation layer, which are calculated as follows:

$$F_{\mathrm{RBL}}\left(X\right)=R\left(X\right)+X$$ (1)

$$R\left(X\right)=\sigma \left(X*W_{1}\right)*W_{2}$$ (2)

where *F_RBL_*(*X*) are the output features of residual block layers, *X* are the input features of residual block layers, and *R*(*X*) is the operation of the convolution layer and the activation function on the input features. *W*_1_ and *W*_2_ are the convolution operations of the 2 convolution layers in a residual block layer. *σ* is the ReLU activation function.

The domain adapter layer in AFAM combines the SENet [35] and attention mechanisms to enable information sharing between different wheat spike features. The domain adapter layer can generate weights sensitive to these features, resulting in more accurate differentiation and representation of wheat spikes at the filling and maturity stages. It adopts the skip connection to process input features: firstly, the features pass through a global average pooling layer, a fully connected layer, and a Softmax activation function to obtain global features; then, 2 branches are designed to process features, considering 2 growth stages adaptively. Finally, weights and new features are generated by channel-wise operation (Eqs. 3 to 6).

$$F_{\mathrm{DAL}}\left(X\right)=F_{1}\left(X\right)*X$$ (3)

$$F_{1}\left(X\right)=F_{2}\left(X\right)*F_{3}\left(X\right)$$ (4)

$$F_{2}\left(X\right)=\upsilon \left(F_{\mathrm{ful}}\right)\left(F_{\mathrm{avg}}\left(X\right)\right)$$ (5)

$$F_{3}\left(X\right)=F_{\mathrm{ful}}\left(\sigma \left(F_{\mathrm{avg}}\left(X\right)\right)\right)$$ (6)

where *F*_DAL_(*X*) are the output features of the domain adapter layer, and *X* are the input features of the domain adapter layer. *F*_avg_ is the global average pooling operation, *F*_ful_ is the fully connected operation, and *υ* is the Softmax activation function.

Color features are important information that significantly reflects differences between wheat spikes at different growth stages [24]. TPGM extracts information such as color and illumination through the Bag of Visual Words Pooling (BVWP), which is a representation of the Bag of Visual Words model in the convolutional network [36], and it can enhance the generalization capability of the model. Input features are combined and mapped to a fixed-length histogram with BVWP:

$${\left[\Phi \left(X\right)\right]}_{k}=\frac{\exp\left(-\left\|X-Y_{k}\right\|/m_{k}\right)}{\sum_{j}\exp\left(-\left\|X-Y_{j}\right\|/m_{j}\right)}$$ (7)

$$H=\frac{1}{N}\sum_{j}\Phi \left(X_{j}\right)$$ (8)

where *X* is the input features, *Y_k_* is the *k*th convolution kernel, exp is the exponential function, and *m_k_* is the scale scaling factor. *N* is the number of the input channels, and *H* is the histogram of feature vectors. TPGM combines a fully connected layer to generate color transformation parameters *α* based on color features and provides transformed wheat spike images to the Detection Network, calculated as:

$$I^{\prime }=\alpha \cdot I$$ (9)

$$\alpha =\nu \left(F_{\text{ful}}\left(X\right)\right)$$ (10)

where *I* is the input image, and *I*^′^ is the new image transformed by the Transform Network fed into the Detection Network. *X* are the input features, *F*_ful_ is the fully connected operation, and *υ* is the Softmax activation function.

### Detection Network

The Detection Network is improved from the standard YOLOv5, which can directly calculate object class, location, and confidence from the wheat spike image. A new microscale detection layer, CSL, and improved localization loss are designed to improve detection accuracy.

#### New microscale detection layer

Three detection layers in the standard YOLOv5 are used to detect objects and output regression and classification [37]. However, small wheat spikes cannot be accurately detected because these 3 layers cannot extract and fuse lower spatial features [27]. Thus, a micro-scale detection layer, one-fourth of the input image size, is added into WheatNet to generate feature maps by extracting lower spatial features and providing more detailed information.

#### CSL for wheat spike angular classification

In this paper, a wheat spike can be labeled as an oriented bounding box (*x*, *y*, *w*, *h*, *cls*, *θ*). (*x*, *y*) refers to the coordinate of the center point. *w* and *h* denote the short and long side lengths of the box. *cls* denotes the category of the object, which is a spike in this study. *θ* is the angle between the direction of the long side and the *x*-axis. WheatNet regards *θ* as the category of angles and introduces CSL [38] (Eq. 11) to measure the angular distance.

$$\mathrm{CSL}\left(x\right)=\left\{\begin{array}{c} g_{a}\left(x\right),\;\theta -\beta <x<\theta +\beta \\ 0,\;\text{otherwise} \end{array}\right.$$ (11)

where *g_a_*(*x*) represents the Gaussian window function. *β* denotes the radius.

#### Optimization for the localization loss

Like the standard YOLOv5, WheatNet has a multi-task loss containing classification, localization, and confidence loss [19]. IoU-based localization loss is only used to determine the degree of overlap area between 2 oriented bounding boxes (Eq. 12). It cannot perform well on the complex spatial relationship among the boxes. Therefore, localization loss is improved by CIoU [39] to achieve higher accuracy:

$$\mathrm{IoU}=\frac{\text{area}\left(A\cap B\right)}{\text{area}\left(A\cup B\right)}$$ (12)

$$\text{CIoU}=1-\mathrm{IoU}+\frac{l\left(O_{A},O_{B}\right)}{d^{2}}+\gamma$$ (13)

where *A* is the anchor box and *B* is the oriented bounding box, respectively. CIoU calculates the Euclidean distance *l*(*O_A_*, *O_B_*) between the center points of *A* and *B*. *γ* can be calculated by the length and width of *A* and *B*.

## Results

### Network settings

Our study conducted the wheat spike detection task in Ubuntu 16 operation system. A workstation (Processor: Intel® Xeon® 8268 CPU; Memory: 500G; Graphics card: NVIDIA Titan V 12G) performed the network training in the deep learning framework PyTorch 1.7. In training, we used Stochastic Gradient Descent for optimization, the batch size was 16, and the learning rate was 0.005. The weight attenuation and momentum were adopted with 1 × 10^−3^ and 0.9, respectively.

### Performance evaluation

In this study, detection accuracy and speed, the 2 main performance metrics, are used to evaluate model performance. Detection speed is measured in frames per second (FPS). Four potential predictions of the oriented detection boxes can be classified. True positive (TP) is the correct detection of wheat spikes. False positive (FP) is the incorrect detection of wheat spikes. False negative (FN) denotes the number of wheat spikes that are not detected. This study does not consider true negative (TN) as the wheat spike detection relies on the foreground. Therefore, precision and recall are calculated as follows.

$$\text{Precision}=\frac{\mathrm{TP}}{\mathrm{FP}+\mathrm{TP}}$$ (14)

$$\text{Recall}=\frac{\mathrm{TP}}{\mathrm{FN}+\mathrm{TP}}$$ (15)

The average precision (*AP*) evaluates the detection accuracy (Eq.16). *AP* reflects the precision rate when the recall rate ranges from 0 to 1. The higher *AP* means the better accuracy of the method.

$$\mathrm{AP}=\int_{0}^{1}\text{Precision}\left(\text{Recall}\right)d\text{Recall}$$ (16)

The angles represent the orientation of wheat spikes in drone imagery, and the accuracy can be calculated by *RMSE_z_* and *MAE_z_* (Eqs. 17 and 18).

$${\text{RMSE}}_{z}=\sqrt{\frac{1}{F}\sum_{i=1}^{F}{\left(p_{i}^{n}-q_{i}^{n}\right)}^{2}}$$ (17)

$${\mathrm{MAE}}_{z}=\frac{1}{F}\sum_{i=1}^{F}\left|p_{i}^{n}-q_{i}^{n}\right|$$ (18)

where *F* represents the number of images, $p_{i}^{n}$ denotes the angle of the predicted box *n* in image *i*, and $q_{i}^{n}$ denotes the angle of the corresponding ground-truth box in image *i*.

The number of detection boxes generated by classical detection methods and WheatNet is used to count wheat spikes. *RMSE_c_*, *rRMSE_c_*, and *MAE_c_* are for evaluation of counting performance (Eqs. 19 to 21).

$${\text{RMSE}}_{c}=\sqrt{\frac{1}{F}\sum_{i=1}^{F}{\left(a_{i}-t_{i}\right)}^{2}}$$ (19)

$${\text{rRMSE}}_{c}=\sqrt{\frac{1}{F}\sum_{i=1}^{F}{\left(\frac{a_{i}-t_{i}}{a_{i}}\right)}^{2}}$$ (20)

$${\mathrm{MAE}}_{c}=\frac{1}{F}\sum_{i=1}^{F}\left|a_{i}-t_{i}\right|$$ (21)

where *t_i_* and *a_i_* denote the number of the predicted spikes and manually labeled spikes in image *i*.

### Wheat spike detection results

#### Performance of WheatNet and other detection methods

**Fig. 4.**
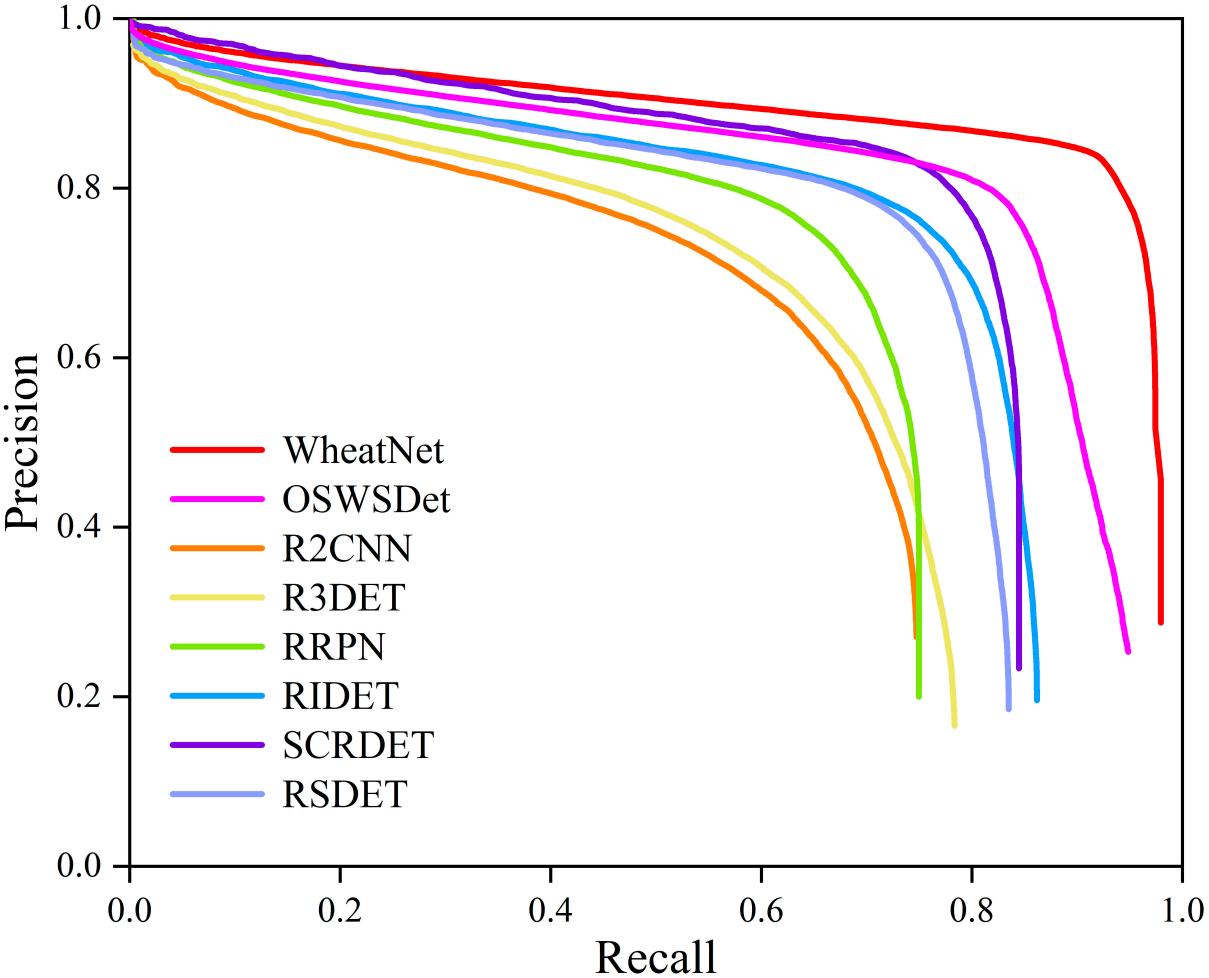
Precision–recall curves of WheatNet and other oriented-object detection methods.

This paper compared WheatNet with classical oriented detection methods [40–45] for wheat spike detection, angle prediction, and speed. WheatNet had *AP*, *RMSE_z_*, and *MAE_z_* of 89.7%, 17.7, and 7.6, respectively (Table 1). The results showed that WheatNet had the highest accuracy for wheat spike detection and accurately described the morphology of wheat spikes. Meanwhile, with precision–recall curves, the precision of the other methods decreased significantly as the recall increased (Fig. 4). When the recall was 0.95, the precision of other oriented detection methods was less than 0.2 and caused serious problems with error detection. In contrast, WheatNet still maintained high precision, and it was a good solution to the problem of miss detection and error detection. The speed of WheatNet is 20 FPS, which allows for rapid and accurate wheat spike detection.

**Table 1.** Performance comparison of WheatNet and oriented-object detection methods.

Model	***RMSE***_***z***_	***MAE***_***z***_	***AP* (%)**	FPS
WheatNet	17.7	7.6	89.7	20
OSWSDet	23.7	10.3	79.4	22
R2CNN	54.8	42.2	59.3	10
R3DET	48.8	38.4	60.8	12
RRPN	49.0	38.9	62.5	10
RIDET	28.7	19.2	72.2	8
SCRDET	26.1	11.1	75.1	13
RSDET	31.3	21.0	69.8	15

**Fig. 5.**
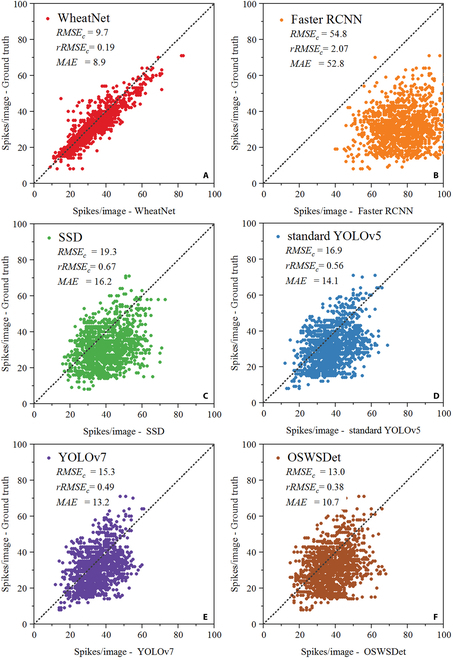
Detection results of WheatNet and other detection methods. (A) WheatNet, (B) Faster R-CNN, (C) SSD, (D) standard YOLOv5, (E) YOLOv7, and (F) OSWSDet.

**Fig. 6.**
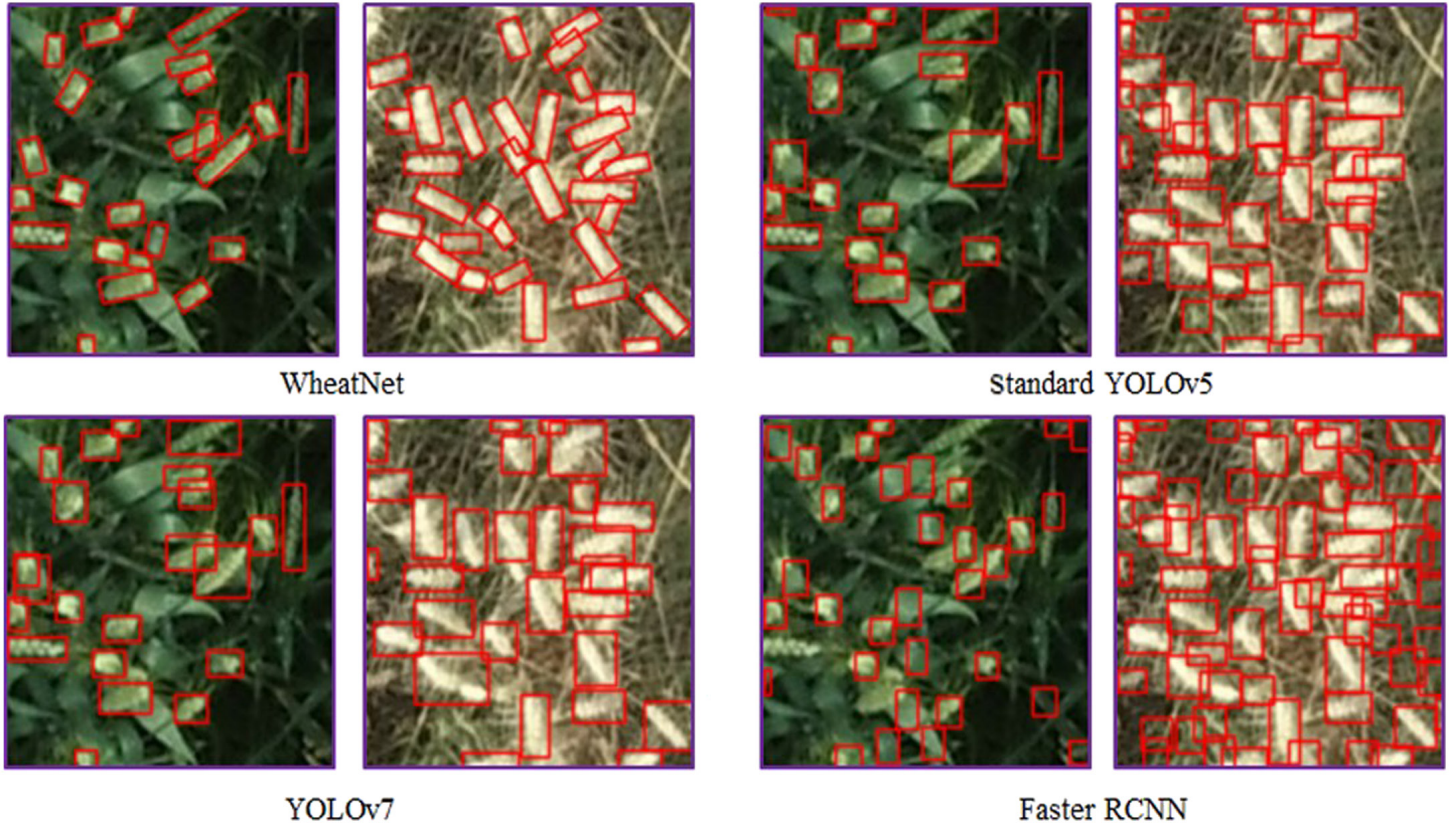
Comparison results of the filling and maturity image detected by WheatNet, standard YOLOv5, YOLOv7, and Faster R-CNN. Red boxes are wheat spike detection boxes.

We also presented a comparison between WheatNet and the classical object detection method as well as the previously developed Oriented and Small Wheat Spike Detection (OSWSDet) [32]. Results showed that the proposed WheatNet outperformed the other methods with the highest accuracy, achieving *RMSE_c_*, *rRMSE_c_*, and *MAE_c_* of 9.7, 0.19, and 8.9, respectively (Fig. 5). Compared with the standard YOLOv5, WheatNet demonstrated a substantial performance improvement, with a reduction of 43% in *RMSE_c_*, 66% in *rRMSE_c_*, and 55% in *MAE_c_*. OSWSDet was constructed in our previous work for a single growth stage, which was improved from the standard YOLOv5 by adopting a CSL, CIoU-based loss, and a micro-scale detection layer. Compared with OSWSDet, *RMSE_c_*, *rRMSE_c_*, and *MAE_c_* of WheatNet reduced by 25%, 50%, and 17%, respectively. The examples of wheat spike detection results with WheatNet, Faster R-CNN, standard YOLOv5, and YOLOv7 are shown in Fig. 6. It can be seen that the WheatNet had excellent performance on wheat spike detection at the filling and maturity stage, and produced fewer error results than other detection methods.

#### Ablation study

In this paper, we investigate the combined effects of a new micro-scale detection layer, a CSL, an improved loss function based on CIoU, and a Transform Network on the detection performance of wheat spikes (Table 2). The Transform Network is the most effective component, leading to an increase in *AP* of 12.9%, and is a viable module to address the problem of inaccurate detection of wheat spikes due to different growth stages. Besides Transform Network, CSL and the microscale detection layer are the 2 most effective modules for accurately detecting small and oriented wheat spikes. These results are consistent with our previous studies [31,32].

**Table 2.** Ablation study of WheatNet on wheat spike detection.

**Circle Smooth Label**	**Micro-scale detection layer**	**CIoU-based localization loss**	**Transform Network**	***AP* (%)**
				51.6
√				63.3
√	√			70.5
√	√	√		76.8
√	√	√	√	89.7

## Discussion

Traditional field survey is inefficient and expensive, so there has been growing interest in image-based techniques for wheat spike detection. Visible images, which can capture color and texture information of objects, are a cost-effective and quickly processed approach for analysis [29,46]. However, it is difficult to mark the same number of wheat spikes from canopy images at the filling and maturity stages due to the field environment and wheat growth [21]. During wheat growth stages, wheat changes significantly in color, size, and morphological features, allowing us to develop key technologies to accurately detect wheat spikes at several critical growth stages [47]. Therefore, it is necessary to develop a deep neural network for wheat spike detection suitable for multiple growth stages to enable accurate yield prediction.

The accuracy of the deep neural network in detecting wheat spikes is affected by large differences in color features of wheat spike images between the filling and maturity stages. Based on our previous research on OSWSDet, we have constructed separate datasets for images captured at the filling and maturity stages to evaluate the performance of OSWSDet. The results demonstrate that the accuracy of wheat spike detection is 90.7% and 90.5% when both the training and test sets are images of the filling or maturity stage, respectively (Table 3). These findings suggest that the accuracy of OSWSDet is high for a single growth stage, which is consistent with the results of previous studies [23,48]. When the training and test sets are from the images of different growth stages, the wheat spike detection accuracy is significantly reduced by 60.0% and 51.6%, respectively (Table 3). This phenomenon suggests that constructing a wheat spike detection model for a single growth stage would not perform well for other growth stages [21].

**Table 3.** *AP* of OSWSDet [28] on different datasets.

**Training dataset**	**Test dataset**	***AP* (%)**
Filling images	Filling images	90.7
Filling images	Maturity images	30.7
Maturity images	Filling images	38.9
Maturity images	Maturity images	90.5

**Fig. 7.**
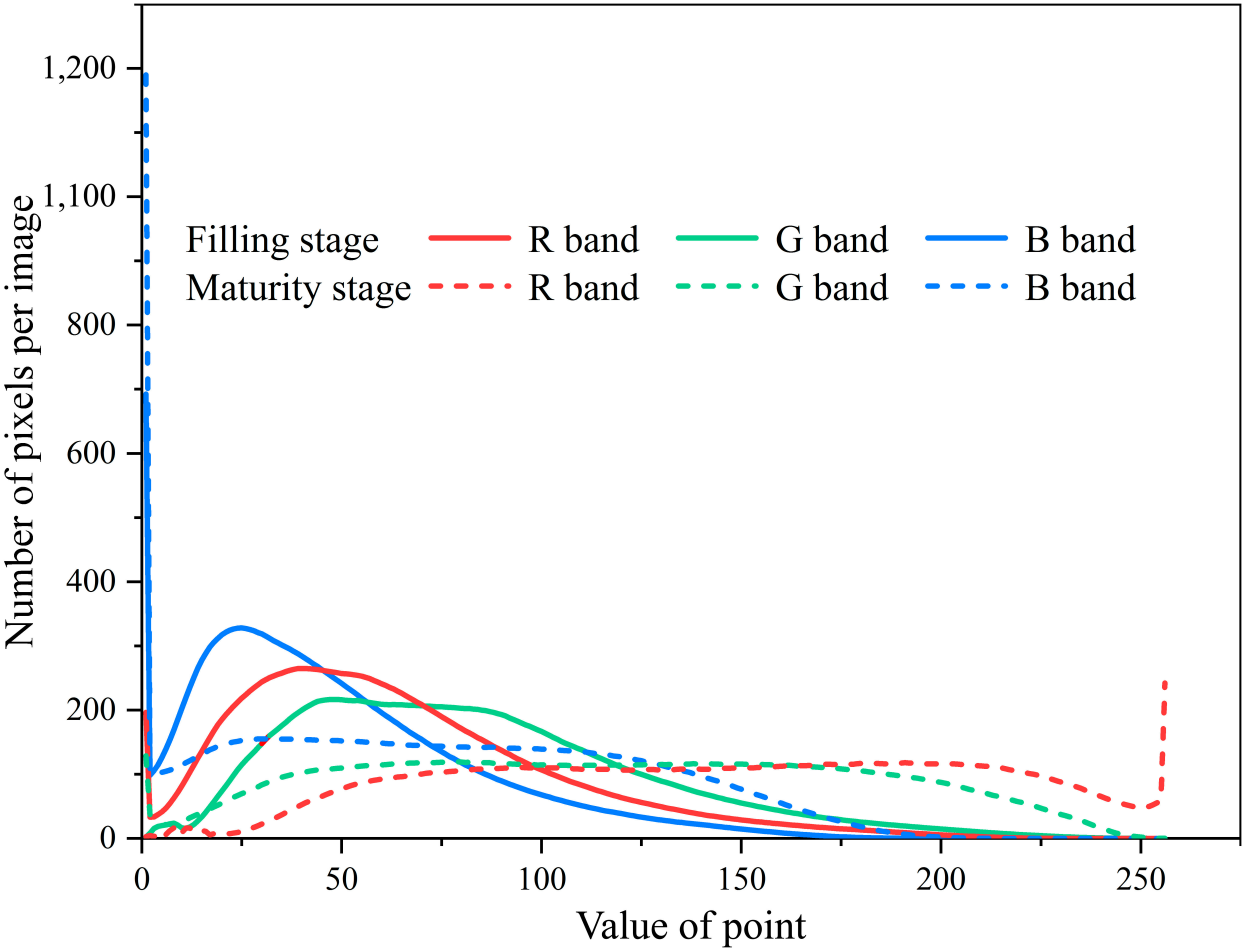
Color histogram for wheat UAV images at the filling stage and the maturity stage.

**Fig. 8.**
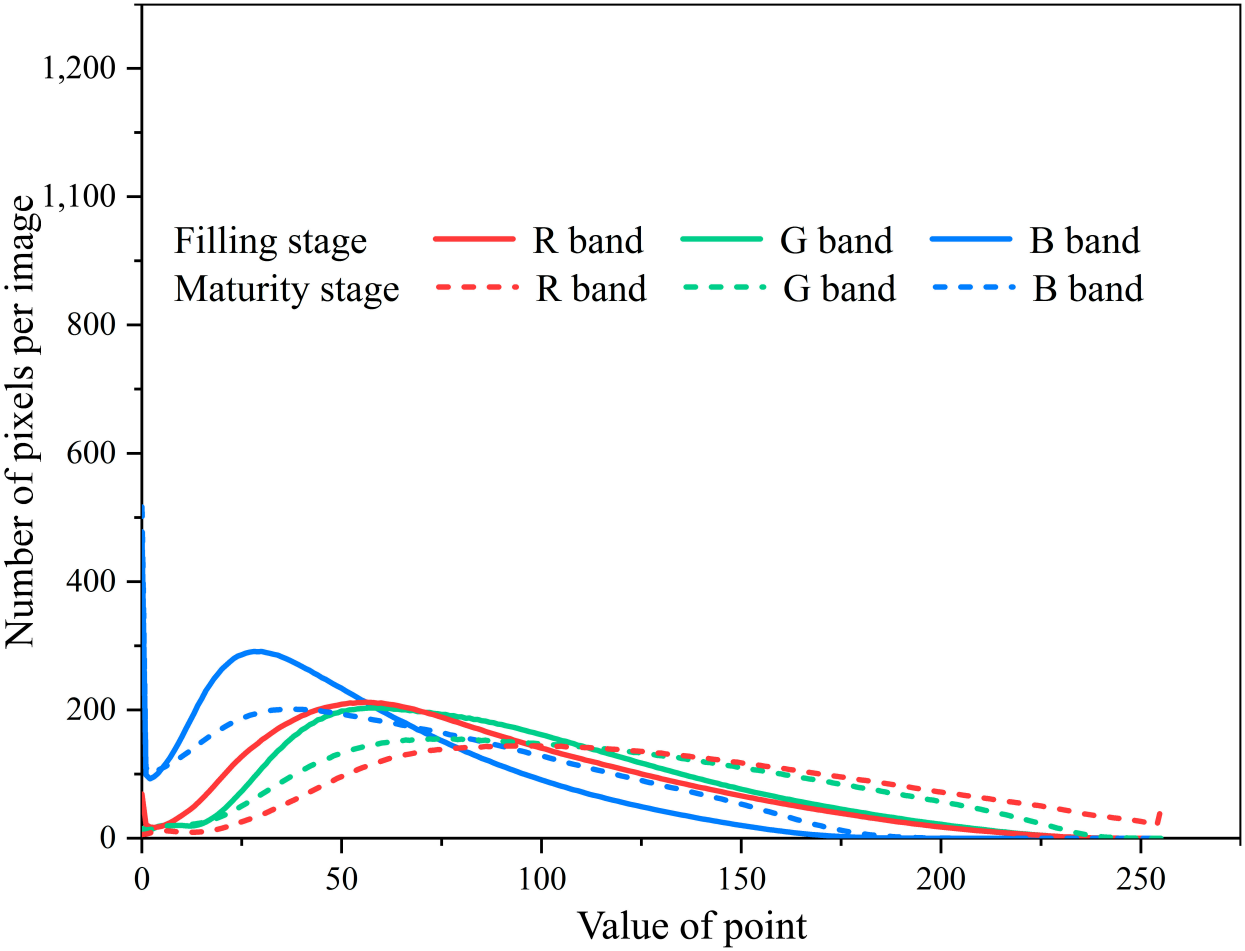
Color histogram for wheat UAV images after transforming.

**Fig. 9.**
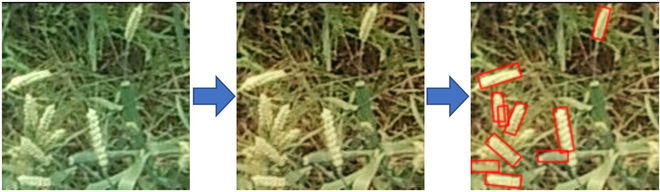
Visualization of detection results and transformed images at the filling stage.

WheatNet effectively reduces the differences in the color features of wheat spike images between the filling and maturity stages. Comparing the new image generated by the Transform Network with the input image (Figs. 7 and 8), the differences in the new image’s R, G, and B bands are significantly reduced. For images taken at the maturity stage, the Transform Network significantly reduces the number of pixel points with pixel values greater than 250 in the red band and less than 5 in the blue band. For example, Fig. 9 shows the results of wheat spike detection at the filling stage. Wheat spikes can be detected accurately in the image transformed by the Transform Network.

WheatNet improves detection accuracy by reducing the difference in features between the wheat spike images at the filling and maturity stages. This paper uses a unified set of wheat spike images at the filling and maturity stages as the training set for the standard YOLOv5 and WheatNet. The performance of these 2 models is evaluated on 3 separate datasets of wheat spike images at the filling stage, at the maturity stage, and at both stages. The results show that even though the wheat spikes are labeled on the images of both the filling and maturity stages, the standard YOLOv5 cannot accurately detect them with an accuracy of only 57.7%, 52.8%, and 55.6%, respectively (Table 4). OSWSDet also only achieves an accuracy of 80.6%, 77.8%, and 79.4%, respectively. There is a higher contrast difference between the wheat spikes and the background at the filling stage compared to the maturity stage. This contrast difference results in the higher accuracy of wheat spike detection at the filling stage [5]. Therefore, constructing a model by directly using wheat spike images at different growth stages does not meet the requirement for accurate detection. WheatNet can reduce detection errors caused by differences in the color features of wheat spikes and be successfully applied to both filling and maturity stages with an accuracy of 90.1% for the filling stage and 88.6% for the maturity image (Table 4).

**Table 4.** *AP* of WheatNet, OSWSDet, and standard YOLOv5 on different test datasets.

**Method**	**Training dataset**	**Test dataset**	***AP* (%)**
WheatNet	Filling and maturity images	Filling images	90.1
WheatNet	Filling and maturity images	Maturity images	88.6
WheatNet	Filling and maturity images	Filling and maturity images	89.7
OSWSDet	Filling and maturity images	Filling images	80.6
OSWSDet	Filling and maturity images	Maturity images	77.8
OSWSDet	Filling and maturity images	Filling and maturity images	79.4
Standard YOLOv5	Filling and maturity images	Filling images	57.7
Standard YOLOv5	Filling and maturity images	Maturity images	52.8
Standard YOLOv5	Filling and maturity images	Filling and maturity images	55.6

Compared to transfer learning, which has been extensively used in previous studies to detect and count wheat spikes [21,25], WheatNet uses color features to improve the performance in detecting wheat spikes at different growth stages. Transfer learning has the advantage that it requires a small amount of training data and that a pre-trained model, trained on a large amount of industrial data, can be quickly and easily applied to field scenes [30]. However, transfer learning uses a single deep neural network that shares all network parameters except the detection layer. It has difficulty in achieving high accuracy when the features of datasets for 2 fields differ significantly [49]. Therefore, transfer learning cannot be applied well to multiple wheat field scenes.

On the other hand, WheatNet is designed to address the problem of accurately detecting wheat spikes over multiple growth stages. While our previous methods, such as OSWSDet [28], have demonstrated high accuracy for a single growth stage, they often perform poorly at other stages (Table 3). WheatNet is an extension of our previous work on OSWSDet and extends the applicability of the wheat spike detection model. As an end-to-end single-stage wheat spike detection model, WheatNet addresses the challenge of applying existing detection methods to multiple growth stages while maintaining detection accuracy.

With prior knowledge, including wheat spike color, morphology, and size features, this study proposes WheatNet for wheat spike detection in UAV images taken at different growth stages. The differences in wheat spike characteristics at different growth stages, particularly color characteristics, affect detection accuracy. Existing wheat spike detection models designed for a single growth stage are difficult to apply to several growth stages or field scenes.

By introducing color features of wheat spikes, WheatNet achieved high accuracy in detecting wheat spikes in UAV images at both the filling and maturity stages, with an *AP* of 89.7%, an *RMSE_c_* of 9.7, an *rRMSE_c_* of 0.19, and an *MAE_c_* of 8.9. With the inherited advantages of OSWSDet, WheatNet can handle the detection of small and oriented wheat spikes in drone imagery. Moreover, it overcomes the limitations of OSWSDet, which can only accurately detect wheat spikes at a single growth stage. It provides valuable insights into wheat spike detection methods for the entire growth stage and has great potential for field applications.

## Data Availability

The authors confirm that the data are available within the article. The data within the article are included in the Supplementary Materials.
